# Perilesional and Contextual Radiomic Features Improve Differentiation of Odontogenic Cysts on Panoramic Radiographs

**DOI:** 10.3390/jcm15155858

**Published:** 2026-07-27

**Authors:** Barbara Obuchowicz, Joanna Zarzecka, Marzena Jakubowska, Rafał Obuchowicz, Julia Lasek

**Affiliations:** 1 Department of Conservative Dentistry with Endodontics, Institute of Dentistry, Jagiellonian University Medical College, Montelupich 4, 31-155 Krakow, Poland; 2 Imaging Diagnostics Laboratory, University Dental Clinic in Krakow, Montelupich 4, 31-155 Krakow, Poland; 3 College of Medical Sciences, 02-678 Warsaw, Poland; 4 Faculty of Geology, Geophysics and Environmental Protection, AGH University of Krakow, 30-059 Krakow, Poland

**Keywords:** odontogenic cysts, radiomics, panoramic radiography, texture analysis, artificial intelligence, machine learning, odontogenic keratocyst, radicular cyst, dentigerous cyst, image-based classification

## Abstract

**Background:** Radiographic differentiation of odontogenic cysts remains challenging due to overlapping imaging features and limited specificity of conventional assessment. Radiomics offers a quantitative approach to extract imaging biomarkers that may improve non-invasive lesion characterization. Accurate differentiation among cyst subtypes is clinically relevant, as different odontogenic cysts may warrant different management and follow-up strategies. **Methods:** This study included 63 patients with histopathologically confirmed odontogenic cysts (odontogenic keratocyst [OKC], *n* = 33; radicular cyst [RC], *n* = 12; dentigerous cyst [DC], *n* = 10; odontogenic cyst, not otherwise specified [OC-NOS], *n* = 8). Panoramic radiographs were manually segmented to define cyst and anatomical regions of interest. Radiomic features were extracted using three groups: agnostic texture features, morphological/intensity descriptors, and perilesional features. Classification was performed using logistic regression with leave-one-out cross-validation. Pairwise and four-class classification analyses were conducted, and feature importance was evaluated. **Results:** Radiomic features demonstrated significant differences between cyst and anatomical regions, with approximately 60% of features remaining significant after correction. Pairwise classification performance varied across lesion types, achieving the highest balanced accuracy for DC vs. OC-NOS (0.838) and OKC vs. OC-NOS (0.752), while comparisons involving OKC vs. RC (0.602) and OKC vs. DC (0.644) were less discriminative. Texture-based features, particularly NGTDM-derived metrics, were among the most informative. The integration of morphological and perilesional features improved performance: four-class balanced accuracy increased from 0.416 with texture-only features to 0.487 with the combined feature set (*p* = 0.002). **Conclusions:** Radiomics-based analysis of panoramic radiographs enables moderate but preliminary differentiation of odontogenic cysts, with performance comparable to prior studies. The inclusion of perilesional and spatial features enhances classification, highlighting the importance of contextual information. Despite these advances, substantial overlap between cyst types persists, underscoring the need for multimodal diagnostic approaches and further validation in larger cohorts.

## 1. Introduction

Odontogenic cysts are a heterogeneous group of lesions arising from remnants of the tooth-forming apparatus, differing in pathogenesis, biological behavior, and clinical presentation. The odontogenic keratocyst (OKC) is a developmental lesion with a propensity for aggressive growth and recurrence, related to its unique epithelial and molecular profile [1]. In contrast, the radicular cyst (RC) is an inflammatory lesion associated with pulpal necrosis, driven by chronic infection and cytokine-mediated epithelial proliferation [2]. The dentigerous cyst (DC) is a developmental lesion linked to impacted or unerupted teeth, resulting from fluid accumulation between the reduced enamel epithelium and the tooth crown [3]. Epidemiologically, radicular cysts are the most common, followed by dentigerous cysts and OKCs, with differences in origin and distribution influencing clinical management [4].

Despite these biological differences, odontogenic cysts often show similar radiographic appearances, typically presenting as well-defined radiolucencies with corticated margins [5,6,7]. The dentigerous cyst usually appears as a unilocular radiolucency surrounding the crown of an unerupted tooth at the cemento–enamel junction, although variability in size and morphology may complicate diagnosis [8,9]. The radicular cyst is most commonly seen as a periapical radiolucency associated with a non-vital tooth [10]. The OKC demonstrates more variable imaging features, ranging from unilocular to multilocular lesions, often in the posterior mandible, with limited buccolingual expansion despite significant anteroposterior growth [11,12].

Radiographic differentiation remains challenging due to overlapping imaging features and non-specific relationships to adjacent teeth [6,7]. Moreover, biological behavior does not consistently translate into distinct radiologic patterns, and factors such as lesion size, infection, and bone remodeling may obscure characteristic findings [13]. Although cone beam computed tomography improves assessment of lesion extent, it does not reliably reflect internal or epithelial characteristics, limiting diagnostic specificity [14]. Consequently, discrepancies between radiographic and histopathological diagnoses are common [15,16]. Emerging quantitative approaches, such as CT texture analysis, show potential but are not yet widely implemented [17], while overlapping epidemiological patterns further complicate interpretation [18].

Overall, the difficulty in differentiating odontogenic cysts radiographically reflects overlapping imaging features, variable biological behavior, and intrinsic limitations of imaging, underscoring the need for a multimodal diagnostic approach.

Radiomics and artificial intelligence (AI) have emerged as promising approaches for improving the diagnostic performance of dentomaxillofacial imaging by enabling quantitative analysis of features not appreciable to the human eye. Radiomics allows extraction of high-dimensional data related to texture, shape, and intensity from standard imaging modalities such as computed tomography and cone beam computed tomography, potentially enhancing differentiation between odontogenic lesions with overlapping visual characteristics [17,19]. Early applications in maxillofacial radiology have demonstrated the feasibility of using machine learning and deep learning models to classify cystic lesions of the jaws, with encouraging diagnostic accuracy [20]. In particular, texture-based analyses have shown potential in distinguishing lesions such as odontogenic keratocysts and other cystic or tumor-like entities, supporting the concept of imaging biomarkers derived from routine radiographic data [21].

More broadly, systematic reviews and conceptual studies highlight the growing role of AI in dental imaging, emphasizing its capacity to support clinical decision-making, reduce diagnostic variability, and augment traditional radiological assessment [22,23]. The integration of neural networks and advanced computational methods into dental radiology reflects a broader shift toward data-driven diagnostics, although challenges related to standardization, validation, and clinical implementation remain [24]. Collectively, these developments suggest that radiomics-based analysis of radiographic images may play an increasingly important role in the non-invasive differentiation of odontogenic cysts, complementing conventional imaging and potentially reducing reliance on invasive diagnostic procedures. Accurate differentiation among odontogenic cyst subtypes is itself clinically relevant, as these lesions differ in biological behaviour, recurrence risk, and management; improved imaging-based characterisation may therefore support more consistent diagnosis and follow-up.

## 2. Materials and Methods

### 2.1. Study Design

This study was designed as a retrospective, single-center observational analysis conducted in accordance with the Declaration of Helsinki and applicable institutional regulations. Ethical approval was obtained from the institutional review board and the Local Bioethics Committee (approval number 106/KBL/OIL/2024, 12 December 2024). Due to the retrospective design and the use of fully anonymized imaging data, the requirement for informed consent was waived.

Radiographic data were retrospectively collected from patients undergoing routine diagnostic evaluation at the University Dental Clinic, Kraków, Poland. The dataset consisted of paired panoramic radiographs (orthopantomograms) and computed tomography examinations (CBCT or CT) acquired for the same anatomical region. Image selection and prescreening were performed by experienced clinicians as part of standard diagnostic workflows. All imaging data were anonymized prior to analysis, and no patient-identifiable information was retained.

The study cohort included adult patients with histopathologically confirmed odontogenic cysts, with both panoramic and cross-sectional imaging available for each case. Inclusion criteria required: (1) availability of matched panoramic and CT/CBCT imaging of the same lesion, (2) adequate image quality with appropriate contrast and absence of significant motion or acquisition artifacts, and (3) clear visualization of lesion boundaries sufficient for segmentation and radiomic analysis. Cases with incomplete imaging, poor-quality scans, or ambiguous lesion margins were excluded.

Additional exclusion criteria comprised the presence of factors that could confound image interpretation or radiomic feature extraction, including extensive metallic artifacts, large restorations obscuring the lesion, prior surgical intervention in the region of interest, or overlap with adjacent anatomical structures that could not be reliably separated. Patients with systemic conditions known to significantly affect bone structure or lesion appearance (e.g., advanced metabolic bone disease or prior head and neck radiotherapy) were also excluded.

A structured screening process was applied to all cases to ensure consistency with inclusion criteria. Where multiple imaging studies were available for a given patient, the examination with the highest diagnostic quality and best lesion visibility was selected. As all data were fully anonymized, detailed demographic variables such as age and sex were not consistently available and were therefore not included in the analysis, in line with the methodological focus of the study.

### 2.2. Study Population and Viewing Methods

All imaging data analyzed in this study were obtained during routine diagnostic procedures between 2022 and 2024 using standard digital radiography systems (KaVo OP 3D Pro) (Instrumentarium Dental, PaloDEx Group Oy, Tuusula, Finland). The study population comprised 63 patients with histopathologically confirmed jaw cysts, retrospectively collected at the University Dental Clinic, Department of Endodontics, Kraków, Poland. Four cyst types were represented: odontogenic keratocyst (OKC; *n* = 33), radicular cyst (RC; *n* = 12), dentigerous cyst (DC; *n* = 10), and odontogenic cyst, not otherwise specified (OC-NOS; *n* = 8). Cohort composition and lesion characteristics are summarised in Table 1.

The fourth group, designated odontogenic cyst, not otherwise specified (OC-NOS), comprised histologically confirmed epithelial-lined odontogenic cysts that lacked the parakeratinized, palisaded epithelial architecture characteristic of OKC and did not meet the combined histopathological and clinicoradiological criteria for radicular or dentigerous cysts. Because radicular and dentigerous cysts may exhibit overlapping, nonspecific non-keratinizing epithelial morphology, their exclusion was based on integration of histopathological findings with tooth vitality, periapical location, and relationship to an unerupted tooth. OC-NOS therefore represents a diagnosis of exclusion and may encompass several distinct entities sharing nonspecific histological features.

The images were stored in DICOM format and evaluated at their original spatial resolution without compression or post-processing that could alter image characteristics.

Image interpretation was performed using high-resolution, medical-grade diagnostic displays (Bracco Imaging S.p.A., Milan, Italy) with 4K ultra-high-definition capability (3840 × 2160 pixels). All assessments were conducted in a dedicated radiology workstation environment equipped with DICOM Part 14-compliant monitors.

Viewing conditions were standardized to ensure optimal image evaluation. Ambient illumination in the reading room was controlled within the range of 25–40 lux to minimize glare and reduce observer fatigue. Basic image manipulation tools, including zoom and contrast adjustment, were available during analysis. Prior to image review, observers were allowed sufficient time to adapt to the ambient lighting conditions. The study was designed to reflect real-world diagnostic scenarios in which radiographic differentiation between cystic lesions and tumor-like entities may be clinically ambiguous.

### 2.3. Radiographic Imaging Protocol

Panoramic radiographs were acquired using a KaVo OP 3D Pro (KaVo Dental GmbH, Biberach, Germany), a digital extraoral imaging system designed for orthopantomographic examinations. All scans were performed according to the manufacturer’s standard clinical protocols for adult patients, with tube voltage settings ranging from 60 to 90 kVp and tube current between 4 and 15 mA, depending on patient size and diagnostic requirements. The exposure time was approximately 10–20 s, corresponding to a full rotational acquisition around the patient’s head. Patients were positioned in a standing posture using standardized head supports and bite blocks to ensure reproducible alignment within the focal trough. Digital panoramic images were reconstructed automatically by the system software and exported in high-resolution format for further analysis. All imaging procedures were conducted in accordance with institutional radiological safety guidelines and optimized to adhere to the ALARA (As Low As Reasonably Achievable) principle.

Images were obtained digitally and stored in DICOM format within the institutional Picture Archiving and Communication System (PACS). The resulting images had a resolution of 1200 × 1600 pixels, with pixel spacing ranging from 0.076 to 0.100 mm (median 0.076 mm; interquartile range 0.076–0.083 mm). All images were analyzed at their original resolution without compression or post-processing.

Cone-beam computed tomography (CBCT) examinations were performed using an Orthopantomograph KaVo OP 3D Pro system (Instrumentarium Dental, PaloDEx Group Oy, Tuusula, Finland), equipped with cephalometric functionality. Patients were positioned in a seated configuration with the use of a bite block and head stabilization devices to minimize motion artifacts. Depending on clinical requirements, a field of view of either 5 × 5 cm or 8 × 15 cm was selected. Image reconstruction was based on acquisitions with an isotropic voxel size of 125 μm during a full 360° gantry rotation. Exposure settings included a tube voltage of 90 kV, a tube current of 6–8 mA, and a scan duration of approximately 10 s.

### 2.4. Analytical Workflow

The analytical framework of this study consisted of several sequential stages designed to ensure methodological consistency and prevent information leakage. First, cases with paired panoramic radiographs and corresponding CT/CBCT examinations were identified and selected based on predefined inclusion criteria.

In the second step, regions of interest (ROIs) were delineated on panoramic images, with lesion localization and labeling informed by cross-sectional imaging findings to ensure accurate anatomical correspondence.

Subsequently, a comprehensive set of radiomic features was extracted from the defined ROIs on panoramic radiographs, including texture-based, morphological, and perilesional descriptors. Feature extraction was performed under standardized conditions using both mask-based and bounding-box region definitions to capture complementary information.

In the final stage, supervised classification was performed using logistic regression as the primary model. Model evaluation was conducted within a cross-validation framework with strict separation of training and testing data, and grouping at the patient level was applied to avoid data leakage between folds.

### 2.5. Lesion Segmentation

All lesion boundaries and anatomical reference structures were manually delineated on panoramic radiographs by a panel of four observers (two dentists and two radiologists, each with at least six years’ clinical experience). These observers did not segment the cases independently; because the panel members had different levels of proficiency with the segmentation software, delineation was performed on a consensus basis. The most experienced annotator produced each contour, which was then jointly reviewed by the remaining observers, who either approved it or proposed adjustments until consensus was reached. Each lesion is therefore represented by a single, consensus-based reference mask. As this workflow yields one agreed segmentation per lesion rather than multiple independent delineations, it was not possible to compute formal interobserver or intraobserver agreement metrics (e.g., Dice/Jaccard overlap, Cohen’s kappa) or the stability of the extracted radiomic features (e.g., intraclass correlation coefficients) for the present cohort. For all cases, the cyst lumen was delineated as label 1. In a subset of cases, an additional ipsilateral anatomical reference ROI composed of adjacent non-pathological cancellous bone was delineated as label 2 to provide a local reference class for feature-level comparison. Segmentation masks were exported in NRRD format and co-registered with the corresponding DICOM files by verifying agreement between stored pixel spacing values (tolerance ±15%).

In patients with multilocular lesions or lesions containing discontinuous radiolucent components, each radiographically distinct cystic component was segmented as a separate ROI when it could be reliably delineated on the panoramic image. Consequently, the number of ROIs exceeded the number of patients in some diagnostic groups, yielding 36 ROIs from 33 OKC patients, 11 ROIs from 10 DC patients, and 67 ROIs from 63 patients overall.

### 2.6. Radiomic Feature Extraction

A structured feature extraction scheme was applied to each ROI under two complementary region-definition modes: mask mode, in which features were computed within the manually delineated contour, and bounding-box mode, in which the tightest axis-aligned rectangle enclosing the mask was used as the analysis region. Three feature groups were computed for each combination of ROI and region mode.

(i) Agnostic radiomic features (33 features per ROI). First-order intensity statistics (13 features), grey-level co-occurrence matrix (GLCM; 6 features), grey-level run-length matrix (GLRLM; 4 features), grey-level size-zone matrix (GLSZM; 4 features), neighbourhood grey-tone difference matrix (NGTDM; 3 features), and grey-level dependence matrix (GLDM; 3 features) were extracted using the PyRadiomics library [25]. Computation used the original, non-filtered image with a fixed bin width of 5 intensity units, intensity normalisation to a scale of 100 with outlier removal at ±3 standard deviations, and B-spline resampling. Shape-based features were extracted separately in 2D using the in-plane pixel spacing. Feature extraction was performed with PyRadiomics version 3.0.1; feature definitions follow the Image Biomarker Standardisation Initiative (IBSI), and the complete extraction parameter file is available from the corresponding author to permit exact reproduction.
(ii) Morphological and intensity features (12 features in mask mode; 8 in bounding-box mode). Contour-based shape descriptors were derived from the segmentation boundary: compactness (isoperimetric ratio), solidity (ratio of contour area to convex hull area), convexity (ratio of convex hull perimeter to contour perimeter), radial-distance coefficient of variation, contour curvature standard deviation, lobulation count (peaks in the smoothed radial distance profile), and scalloping index (excess perimeter relative to the convex hull). Boundary sharpness was characterised by the mean, standard deviation, and 90th percentile of the Sobel gradient magnitude sampled along the segmentation contour. Rim contrast was quantified as the mean and 90th percentile of pairwise absolute intensity differences between a 0.6 mm inner shell and a 0.9 mm outer ring around the contour. In bounding-box mode, shape descriptors were omitted because a rectangle has trivially defined geometry; instead, mean, standard deviation, and range of pixel intensities within the box, together with border and interior gradient statistics, were computed.
(iii) Perilesional features (13 features). The immediate peri-lesional tissue was characterised by computing pixel statistics within a morphological dilation shell of 1.5 mm around the ROI boundary: mean intensity, standard deviation, and Shannon entropy of the ring; the ratio and absolute difference of mean intensities between the lesion interior and the ring; the shell-to-core intensity ratio (interior shell of 0.9 mm erosion versus residual core); and the ratio of mean gradient magnitude in the inner shell to that in the outer ring. Spatial context was encoded by the normalised centroid coordinates (relative to image dimensions), the aspect ratio and area (mm^2^) of the bounding box, and the normalised lesion extent along horizontal and vertical image axes. In bounding-box mode, the dilation shell and erosion core were implemented as rectangular approximations preserving equivalent physical dimensions.

A combined feature set (semantic + perilesional, denoted Sem+Peri) was constructed by concatenating the morphological/intensity and perilesional feature vectors for cases present in both sets, resulting in up to 25 features per ROI. All feature sets were computed independently for cyst and anatomical ROIs.

### 2.7. Statistical Analysis and Classification

All classification experiments followed a fully pre-specified analysis plan established before any model results were examined, in accordance with transparent reporting guidelines. No post-hoc model selection was performed. For analyses requiring one observation per case, each case was represented by the largest segmented cystic component.

The primary classifier was logistic regression with L2 regularisation (*C* = 0.1, balanced class weights), designated as the primary model for all inferential conclusions. A linear support vector machine (SVM; *C* = 0.1, balanced class weights) was evaluated as a secondary, exploratory comparator; its results are reported in full but do not support primary inferences.

Model performance was estimated using leave-one-out cross-validation (LOOCV). Within each fold, the following steps were applied exclusively to the training partition to prevent information leakage: (1) standardisation of all features to zero mean and unit variance; (2) removal of features with a coefficient of variation ≤ 0.01 (near-constant features); and (3) for feature sets exceeding 10 features, univariate filter-based selection of the top *k* = 5 features ranked by one-way ANOVA *F*-statistic. Feature sets containing ≤10 features were used without further selection.

The primary performance metric was balanced accuracy (the arithmetic mean of per-class recall), which accounts for class imbalance without requiring resampling. Ninety-five percent confidence intervals were derived by stratified bootstrap resampling of the LOOCV predictions (2000 iterations). Statistical significance was assessed by a label-permutation test (500 permutations) in which the full LOOCV procedure, including feature selection, was repeated on each permuted label vector; the reported p-value equals the proportion of permuted balanced accuracies equal to or exceeding the observed value. Permutation testing was applied to the primary model only. All statistical tests were two-sided, and a *p*-value < 0.05 was considered statistically significant.

### 2.8. Cyst Versus Anatomical Region Analysis

To characterise the feature-level discriminability between cyst and anatomical reference ROIs from the same patient, each radiomic feature was tested individually. For cyst-versus-anatomical comparisons, each ROI type was represented by the largest ROI per case. Paired comparisons were performed only when both ROI types were available for the same case and at least 5 matched case pairs were present; otherwise, unpaired comparisons were used. Effect size was quantified by Cohen’s d. Multiple comparisons within each feature set were controlled using the Benjamini–Hochberg procedure at a false discovery rate of 5%. All statistical tests were two-sided.

### 2.9. Pairwise and Multiclass Classification

Pairwise binary classification was performed for all six combinations of the four cyst types. Multiclass four-way classification was evaluated using two strategies: a direct approach, in which the classifier was trained on all four classes simultaneously, and a hierarchical approach, in which a first-stage binary classifier separated odontogenic keratocysts (OKC) from all remaining types, followed by a second-stage three-class classifier applied to non-OKC cases. The hierarchical decomposition was motivated by the distinct biological behaviour of OKCs relative to the other cyst types, given their developmental origin and propensity for recurrence. Feature importance was assessed post hoc by computing, for each feature and pairwise class comparison, the area under the receiver operating characteristic curve (AUC) and Cohen’s d, with multiple-comparison correction applied across features within each pair. The same evaluation framework was applied to both radiomics-based models and human observer assessments to ensure comparability of results.

### 2.10. Human Reader Evaluation

To provide a reference benchmark for radiomics-based classification, a structured human reader study was conducted using the same set of panoramic radiographs included in the primary analysis.

A total of four observers participated in the study: two dentomaxillofacial radiologists and two dentists, each with a minimum of 6 years of clinical experience in dental imaging. All readers were familiar with panoramic radiographic interpretation in routine clinical practice. Image evaluations were performed independently, and no consensus reading was used at any stage of the analysis.

All observers were blinded to histopathological diagnosis, clinical data, and results of radiomic analysis. Cases were presented in a randomized order, and each observer assigned a single diagnosis per case, selecting one of four categories: odontogenic keratocyst (OKC), radicular cyst (RC), dentigerous cyst (DC), or odontogenic cyst (OC-NOS).

Image review was conducted using the same standardized workstation environment described in Section 2.2, including high-resolution DICOM-calibrated diagnostic displays (4K resolution) and controlled ambient lighting conditions (25–40 lux). Observers were allowed to use basic image manipulation tools (zoom, windowing, and contrast adjustment), consistent with routine diagnostic practice. No explicit time constraints were imposed; however, readers were instructed to perform evaluations under typical clinical conditions.

To assess intra-reader agreement, a subset of cases (randomly selected 20% of the dataset) was re-evaluated by each observer after a washout period of at least 4 weeks, minimizing recall bias. Repeat readings were performed under identical conditions and without access to prior responses.

The reference standard (ground truth) for all cases was based on histopathological diagnosis obtained from surgical specimens, as described in Section 2.1. Human reader performance was evaluated at the case level, with each case contributing a single classification label. Statistical analysis included balanced accuracy, class-specific sensitivity and specificity (one-vs-rest approach), and agreement metrics (Cohen’s kappa for interobserver and intraobserver variability), ensuring direct comparability with the radiomics-based classification framework.

## 3. Results

### 3.1. Cohort Characteristics

**Figure 1:**
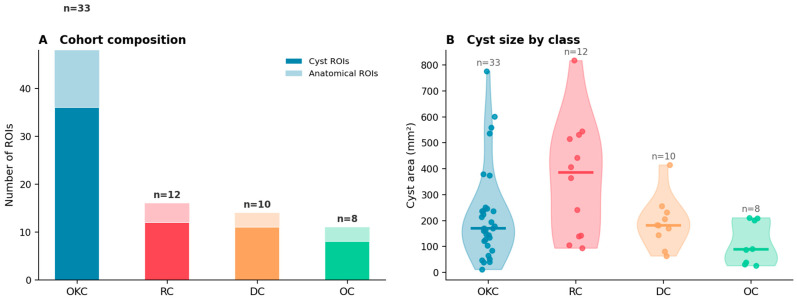
Cohort composition and cyst size distributions. (**A**) Segmented ROI counts per cyst type; numbers above bars denote unique patients per class. (**B**) Cross-sectional cyst area (mm^2^) per class; horizontal lines represent medians and individual data points are superimposed with jitter. OKC = odontogenic keratocyst; RC = radicular cyst; DC = dentigerous cyst; OC-NOS = odontogenic cyst.

The final cohort comprised 63 patients contributing 96 segmented ROIs (67 cyst, 29 anatomical reference). Cyst cross-sectional area differed across types (Kruskal–Wallis; *p* = 0.038). Radicular cysts exhibited the largest median area (385.3 mm^2^; mean ± SD: 362.0 ± 223.8 mm^2^), followed by dentigerous cysts (181.1 mm^2^; 185.1 ± 97.4 mm^2^), keratocysts (168.9 mm^2^; 215.3 ± 173.5 mm^2^), and odontogenic cysts (88.6 mm^2^; 111.9 ± 82.5 mm^2^). Cyst ROIs were substantially larger than anatomical reference ROIs (Mann–Whitney U, *p* < 0.001; Cohen’s d = 1.56). Cohort composition and size distributions are shown in Figure 1.

### 3.2. Radiomic Feature Differences Between Cyst and Anatomical Regions

**Figure 2:**
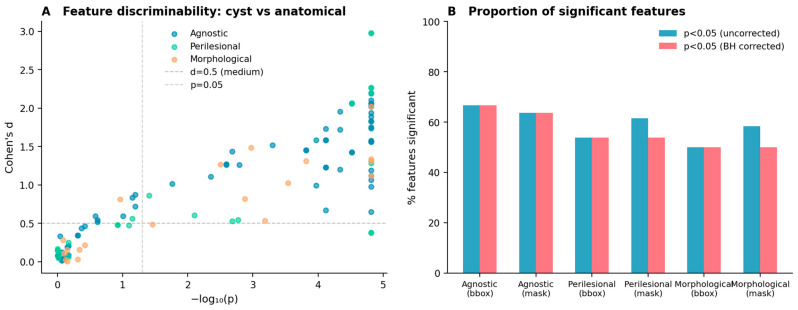
Feature-level discrimination between cyst and anatomical regions. (**A**) Effect size (Cohen’s d) plotted against −log10(*p*) for each feature, coloured by feature category. Dashed reference lines indicate *p* = 0.05 and d = 0.5 (medium effect size [26]). (**B**) Proportion of features reaching statistical significance before and after Benjamini–Hochberg correction, stratified by feature category and region-definition mode.

To establish whether the extracted features carried lesion-specific information, cyst and anatomical ROIs from the same patient were compared across all feature sets and region modes (112 feature–set combinations in total). Sixty-nine features (61.6%) showed a statistically significant difference at an uncorrected threshold of *p* < 0.05; 67 (59.8%) remained significant after Benjamini–Hochberg correction (Figure 2). The agnostic feature set resulted in the highest proportion of discriminating features (21–22 of 33, regardless of region mode), reflecting the sensitivity of texture-based descriptors to the fluid-filled, homogeneous appearance of cyst lumens relative to surrounding bone or soft tissue. Morphological features reached significance in 6–7 of 12 (mask mode) or 4 of 8 (bounding-box mode) instances, while perilesional features were significant in 7–8 of 13 comparisons. These findings confirm that the feature representation captures meaningful lesion-specific signals warranting classification experiments.

### 3.3. Pairwise Binary Classification

**Figure 3:**
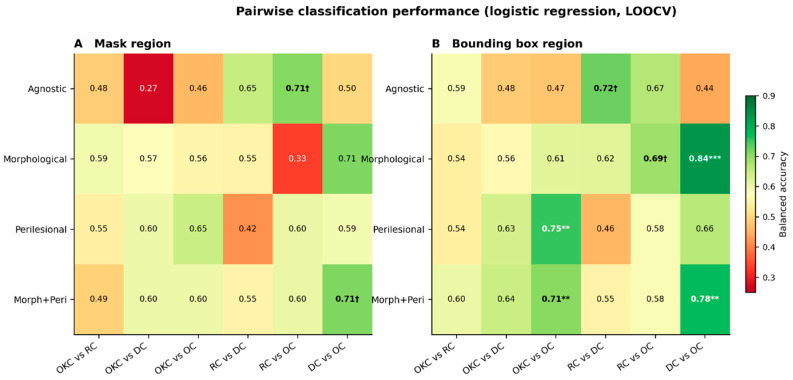
Pairwise binary classification performance. Balanced accuracy of logistic regression (LOOCV) for each feature set and class pair in mask (**A**) and bounding-box (**B**) region modes. Significance symbols reflect permutation test results: † *p* < 0.10, ** *p* < 0.05, *** *p* < 0.01 (500 permutations, primary model only).

Pairwise LOOCV classification was performed for all six combinations of the four cyst types, yielding 48 condition-specific experiments (four feature sets × two region modes × six pairs). Classification performance varied substantially across class pairs (Figure 3, Table 2).

The most discriminable comparison was DC versus OC-NOS: morphological bounding-box features achieved a balanced accuracy of 0.838 (95% CI: 0.662–1.000; sensitivity 0.875, specificity 0.800; permutation *p* = 0.004). This result is consistent with the pronounced difference in lesion location and morphology between dentigerous and odontogenic cysts. The OKC versus OC-NOS comparison was best classified by perilesional bounding-box features (balanced accuracy 0.752, 95% CI: 0.564–0.922; *p* = 0.018), with high specificity (0.879), suggesting that spatial and contextual features capture the distinct osseous environment of keratocysts. Agnostic features performed best for RC versus DC (balanced accuracy 0.725, 95% CI: 0.542–0.900; *p* = 0.056) and RC versus OC-NOS (balanced accuracy 0.708, 95% CI: 0.500–0.896; *p* = 0.050), indicating that intensity texture is informative for differentiating the inflammatory-origin radicular cyst from the remaining types. Pairs involving OKC versus RC and OKC versus DC were the most challenging; no feature set achieved permutation significance for these comparisons, likely reflecting overlapping radiographic appearances among the most prevalent cyst types. The bounding-box region mode matched or exceeded the mask-mode performance in the majority of experiments, suggesting that the immediate peri-contour context contributes discriminative information beyond the lesion interior. Because the OC-NOS group comprised only eight cases, comparisons involving this category are accompanied by wide confidence intervals and should be regarded as exploratory.

### 3.4. Feature Importance

**Figure 4:**
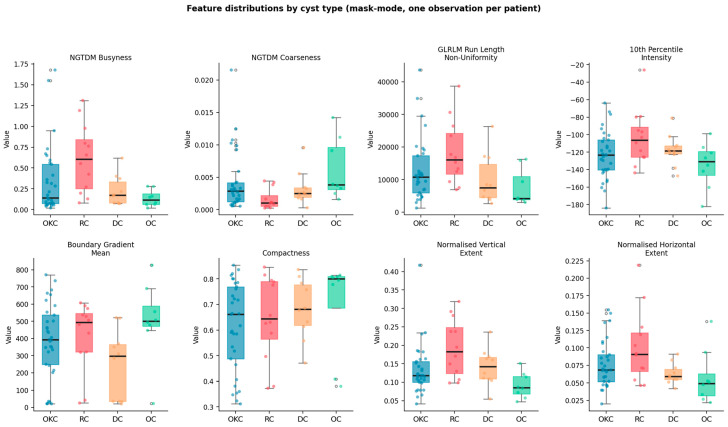
Distributions of the eight highest-ranked radiomic features by cyst type (mask-mode, one observation per patient after deduplication). Box plots indicate medians and interquartile ranges; individual observations are overlaid with random horizontal jitter. Features are ordered by average cross-pair AUC (descending). OKC = odontogenic keratocyst; RC = radicular cyst; DC = dentigerous cyst; OC-NOS = odontogenic cyst.

Univariate discriminability was evaluated for each feature across all six pairwise class comparisons using mask-mode, deduplicated cyst ROIs. No feature attained Benjamini–Hochberg significance in any individual comparison, consistent with the limited statistical power imposed by group sizes of 8–33 cases. Nevertheless, rank-ordering by average AUC across pairs identified a consistent hierarchy of informative features (Table 3, Figure 4).

The highest-ranked features were perilesional spatial descriptors and NGTDM texture measures. Normalised vertical extent of the bounding box (average AUC 0.730, average d = 0.872) ranked first, reflecting systematic differences in the inferosuperior dimension of cysts relative to image size across types. NGTDM busyness and coarseness (average AUC 0.711 and 0.703, respectively) ranked among the top five agnostic features, capturing differences in the local intensity heterogeneity that distinguishes the fluid contents of cyst types with differing epithelial linings. Boundary gradient mean (average AUC 0.673) was the highest-ranked morphological feature, consistent with the sharper corticated margins typically associated with odontogenic cysts and keratocysts relative to inflammatory lesions. The rankings were stable across region modes.

### 3.5. Four-Class Multiclass Classification

**Figure 5:**
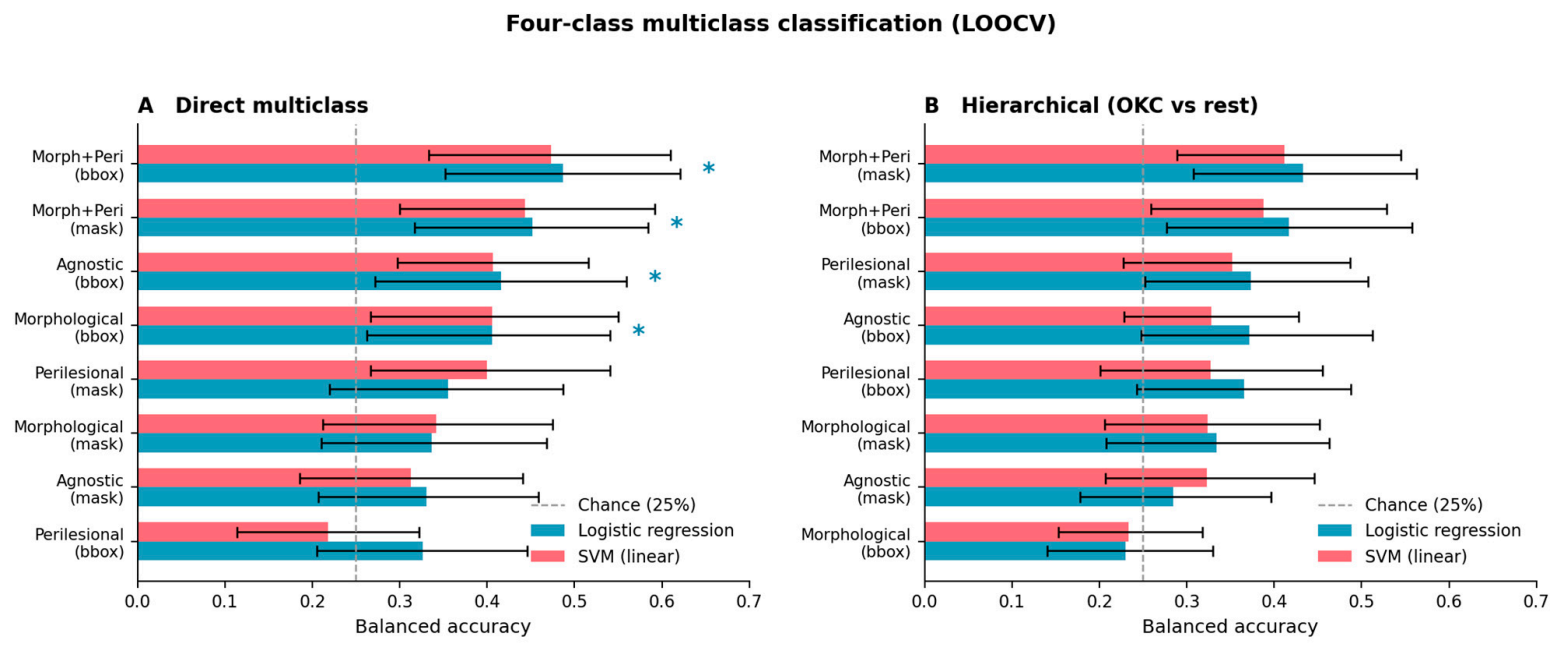
Four-class multiclass classification performance (LOOCV). Balanced accuracy and bootstrap 95% confidence intervals are shown for logistic regression (blue) and linear SVM (red) across all feature sets. The dashed line denotes chance level (0.250). Asterisks indicate permutation significance at *p* < 0.05 for the primary logistic regression model. (**A**) Direct four-class strategy. (**B**) Hierarchical strategy (binary keratocyst versus rest followed by three-class).

Four-class LOOCV was performed across all feature sets and both region modes using direct and hierarchical classification strategies (Table 4, Figure 5). Under the direct strategy, the combined morphological and perilesional feature set in bounding-box mode achieved the highest balanced accuracy of 0.487 (95% CI: 0.352–0.621; permutation *p* = 0.002), representing a 95% improvement over the chance level of 0.25. The corresponding mask-mode combined set results in a balanced accuracy of 0.452 (*p* = 0.012). The agnostic bounding-box features also achieved above-chance performance (balanced accuracy 0.416; *p* = 0.016), confirming that texture information alone carries a multi-class discriminative signal. By contrast, the single-category feature sets (morphological or perilesional alone) did not reach permutation significance, underscoring the complementarity of the two feature groups.

The hierarchical strategy, which isolated keratocysts in a first binary stage before applying a three-class classifier to the remaining types, did not consistently improve upon direct four-class classification. Hierarchical balanced accuracies were generally lower or comparable to the direct approach across all feature sets, suggesting that the single-stage classifier can implicitly learn the keratocyst-separating boundary without explicit decomposition. Performance of the linear SVM was broadly comparable to logistic regression, with overlapping confidence intervals in all conditions, supporting the robustness of the findings to classifier choice. These findings indicate that the model captures a meaningful diagnostic signal, although class overlap limits complete separation between lesion types.

To interpret the added value of the contextual features, the perilesional descriptors were partitioned into location-related subsets (centroid position; and a broader spatial block additionally including normalised extent, bounding-box aspect ratio and area) and a peri-lesional tissue subset (ring, shell and boundary-gradient intensities), each evaluated under the identical LOOCV protocol. Location-related features alone did not demonstrate statistically reliable above-chance discrimination in this cohort (balanced accuracy 0.223–0.284; permutation *p* ≥ 0.26), whereas the peri-lesional tissue subset alone remained significant (0.387; *p* = 0.040). Lesion size, which differed between diagnostic groups, was a substantial discriminator on its own (area-only balanced accuracy 0.430; *p* = 0.002); conversely, removing all explicit size, extent and positional features from the combined morphological–perilesional set left a significant residual signal (0.421; *p* = 0.022). The size-only model failed to resolve the intermediate-sized keratocysts and dentigerous cysts (keratocyst recall 0.21), which were recovered by the combined model (keratocyst recall 0.42; overall balanced accuracy 0.487).

### 3.6. Human Reader Evaluation and Statistical Modeling

To provide a reference benchmark for radiomics-based classification, diagnostic performance of human observers (radiologists and dentists) was evaluated using a structured reader study design. Observers independently reviewed panoramic radiographs and assigned each case to one of four diagnostic categories: odontogenic keratocyst (OKC), radicular cyst (RC), dentigerous cyst (DC), or odontogenic cyst (OC-NOS).

Observer performance was quantified as a multiclass classification task, and results were summarized using balanced accuracy, defined as the average of per-class recall to account for class imbalance. In addition, class-specific sensitivity and specificity were calculated using a one-vs-rest approach, whereby each lesion type was treated as the positive class against all remaining categories. For overall lesion detection (cyst versus non-cyst), human observers achieved a sensitivity of 88%, indicating a high ability to identify the presence of pathology.

To ensure comparability with the radiomics model, evaluation was performed at the case level, with each case contributing a single prediction. Overall performance of human observers reached approximately 45% balanced accuracy, exceeding the chance level of 25% for four-class classification but indicating limited discriminative ability across lesion types. In contrast, intra-reader raw agreement reached 78% (Cohen’s κ 0.59–0.70; Table 5), reflecting relatively good consistency in repeated assessments despite reduced accuracy in detailed subtype classification.

Agreement between observers and the reference standard (histopathological diagnosis) was further assessed using confusion matrix analysis, allowing identification of systematic misclassification patterns. Interobserver variability was quantified using Cohen’s kappa coefficient, providing a measure of agreement beyond chance.

This statistical framework enables direct comparison between human and radiomics-based classification, highlighting differences in performance, error structure, and consistency across diagnostic categories. This comparison was performed under identical evaluation conditions to ensure methodological consistency. This indicates that while observers were highly effective in detecting the presence of lesions, differentiation between cyst subtypes remained limited.

Reader-specific balanced accuracy ranged from approximately 0.42 to 0.48, with a mean value of 0.45 across the four observers (Table 5). When calculated using a one-vs-rest approach and macro-averaged across the four diagnostic categories, sensitivity corresponded to the reader-specific balanced accuracy and ranged from 0.42 to 0.48, whereas specificity was higher, ranging from approximately 0.80 to 0.83. Intra-reader agreement was moderate to substantial, with Cohen’s κ values ranging from approximately 0.59 to 0.70. Agreement among all four readers was fair, with an estimated interobserver κ of approximately 0.30.

### 3.7. Comparison of Human and Radiomics-Based Classification Performance

Compared to human observer performance, the radiomics-based model achieved broadly similar discrimination. Readers achieved a macro-averaged one-vs-rest specificity of 0.80–0.83 in the four-class setting (Table 5), while the radiomics model reached specificities of 0.80–0.88 in pairwise comparisons and 0.67–0.79 in more challenging scenarios. These figures are not directly comparable: reader specificity was computed one-vs-rest for the four-class task, whereas the higher radiomics specificities are drawn from pairwise sub-problems, and no formal statistical test was performed between readers and models. The values are therefore descriptive only and should not be read as evidence that radiomics outperforms expert readers.

In the multiclass framework, which is inherently more demanding, chance-level performance is 25% for four categories. The radiomics model achieved a balanced accuracy of 0.487, representing nearly a twofold improvement over chance. Human readers, evaluated on the same four-class task, reached approximately 45% balanced accuracy, which is likewise modest and close to the model’s value.

Together, these findings suggest that although the radiomics model successfully captures diagnostically relevant signal and performs at a level broadly comparable to expert human assessment, misclassification between closely related cyst types remains substantial, highlighting both the complexity of the task and the need for further methodological refinement.

## 4. Discussion

### 4.1. Radiomics Performance in Odontogenic Lesion Differentiation

The present results are in line with prior radiomics studies demonstrating moderate but meaningful discriminative performance in the differentiation of odontogenic and periapical lesions. In our cohort, pairwise classification achieved balanced accuracies up to 0.838 for DC vs. OC-NOS and 0.752 for OKC vs. OC-NOS, while more challenging comparisons such as OKC vs. RC (0.602) and OKC vs. DC (0.644) remained non-significant, reflecting substantial feature overlap. A similar pattern was reported by Oda et al. [17], where CT texture analysis enabled differentiation of odontogenic cysts but with moderate accuracy and overlap between lesion types, particularly in clinically similar entities. Their subsequent work on broader cystic lesions also demonstrated limited separability across certain classes, consistent with our findings.

### 4.2. Comparison with Radiomics Studies in Periapical Lesions

Studies focusing on periapical lesions report comparable performance ranges. De Rosa et al. [19] showed that CBCT texture analysis could differentiate radicular cysts from granulomas, but with overlapping feature distributions, while Pociask et al. [27] reported classification accuracies around 70–80%, closely matching our results for RC-related comparisons (balanced accuracy 0.708–0.725). These consistencies support the notion that texture-based radiomics captures relevant but not fully discriminative information in inflammatory and cystic lesions.

### 4.3. Role of Texture Features in Lesion Characterization

From a feature perspective, our study identified NGTDM-based heterogeneity metrics (busyness, coarseness) and non-uniformity measures as top-ranked descriptors, which directly aligns with Oda et al. [17] and Gomes et al. [21], both of whom emphasized the importance of texture heterogeneity in reflecting underlying tissue composition. Similarly, Gomes et al. [21] demonstrated that MRI texture features could differentiate OKC from ameloblastoma with good performance, although not without overlap, reinforcing the modality-independent value of radiomics features.

### 4.4. Added Value of Perilesional and Contextual Features

Importantly, our findings extend previous work by demonstrating that perilesional and morphological features contribute to classification performance. The best-performing models in our study relied on combined semantic and perilesional features (balanced accuracy 0.487 in four-class classification), outperforming texture-only models. This contrasts with earlier studies such as De Rosa et al. [19] and Pociask et al. [27], which primarily focused on intralesional texture, and suggests that contextual imaging information may capture additional biologically relevant differences. This contribution did not act through lesion position, as normalised spatial coordinates and extent were non-discriminative in isolation. Lesion size, which differed between groups, was itself a strong discriminator but separated only the size-extreme classes—the largest (radicular) and smallest (odontogenic) cysts—and not the mutually similar, intermediate-sized keratocysts and dentigerous cysts. Notably, after all explicit size and positional information was removed, the imaging descriptors still discriminated the four types significantly. These observations also bear on the role of lesion localisation. In routine practice, differential diagnosis relies heavily on semantic anatomical relationships—periapical position, association with the crown of an impacted tooth, or contact with specific structures. Such relationships were not available as structured per-lesion annotations in this retrospective, anonymised cohort, and were deliberately not hand-engineered as categorical inputs, because they largely encode the visual diagnostic criteria themselves and would conflate the quantitative-imaging signal we sought to isolate with human pattern recognition. Consistent with this, the low-level positional descriptors that were available (normalised centroid and extent) classified the four types only at chance level. A hybrid model coupling radiomic descriptors with structured anatomical-relationship features is therefore a promising direction that could improve sensitivity and specificity, and represents a clear next step. As no odontogenic tumors were included in the present cohort, these findings apply only to differentiation among the studied cyst categories; extension to cyst–tumor discrimination would require dedicated cohorts containing neoplastic lesions. A similar trend toward improved performance with comprehensive radiomic feature sets has been observed in more recent CBCT-based work by Lopes et al. [28].

### 4.5. Overall Performance and Limitations of Radiomics Approaches

Overall, the consistency between our results and prior studies—both in terms of performance ranges (typically 0.6–0.8 balanced accuracy) and the importance of texture heterogeneity features—supports the robustness of radiomics for odontogenic lesion differentiation, while also confirming its current limitations in resolving closely related cyst subtypes.

### 4.6. Advances in AI and Deep Learning Approaches

Beyond classical radiomics, several studies have explored advanced artificial intelligence frameworks integrating machine learning and deep learning, often reporting improved or more scalable diagnostic performance. These hybrid approaches leverage automated feature selection and nonlinear decision boundaries, which may better capture complex imaging patterns than linear models such as those used in the present study. Deep learning methods further extend this paradigm by enabling end-to-end image analysis without predefined feature extraction, demonstrating promising diagnostic accuracy in dentomaxillofacial imaging tasks.

### 4.7. Clinical Applicability and Practical Considerations

However, while these advanced approaches often report higher performance, they typically require larger datasets, extensive training, and external validation, which may limit their immediate clinical applicability. In contrast, our approach—based on interpretable radiomic and contextual features—offers a transparent and data-efficient alternative, particularly suitable for smaller, well-characterized cohorts. Together, these findings suggest that while deep learning and hybrid AI frameworks represent the next step in automated lesion classification, radiomics-based methods remain valuable, especially when interpretability and limited data are key considerations.

### 4.8. Limitations of Conventional Radiographic Assessment

Finally, conventional clinicoradiological approaches continue to demonstrate important limitations in the differentiation of odontogenic lesions, providing a benchmark against which AI-based methods can be evaluated. Banomyong et al. [29] reported a significant overlap between clinical, radiographic, and histopathological diagnoses of periapical lesions, underscoring the limited specificity of traditional imaging criteria and the risk of misclassification in routine practice. Similarly, de Souza et al. [30] highlighted that, despite advances in imaging, accurate discrimination between odontogenic cysts and tumors remains challenging without adjunctive analytical tools.

These findings reinforce the need for quantitative and AI-driven approaches, such as radiomics, to improve diagnostic precision. In this context, the present study contributes to bridging the gap between conventional radiological assessment and advanced computational analysis, supporting the integration of data-driven methods to overcome the inherent limitations of visual interpretation.

### 4.9. Human Observer Performance and Diagnostic Challenges

**Figure 6:**
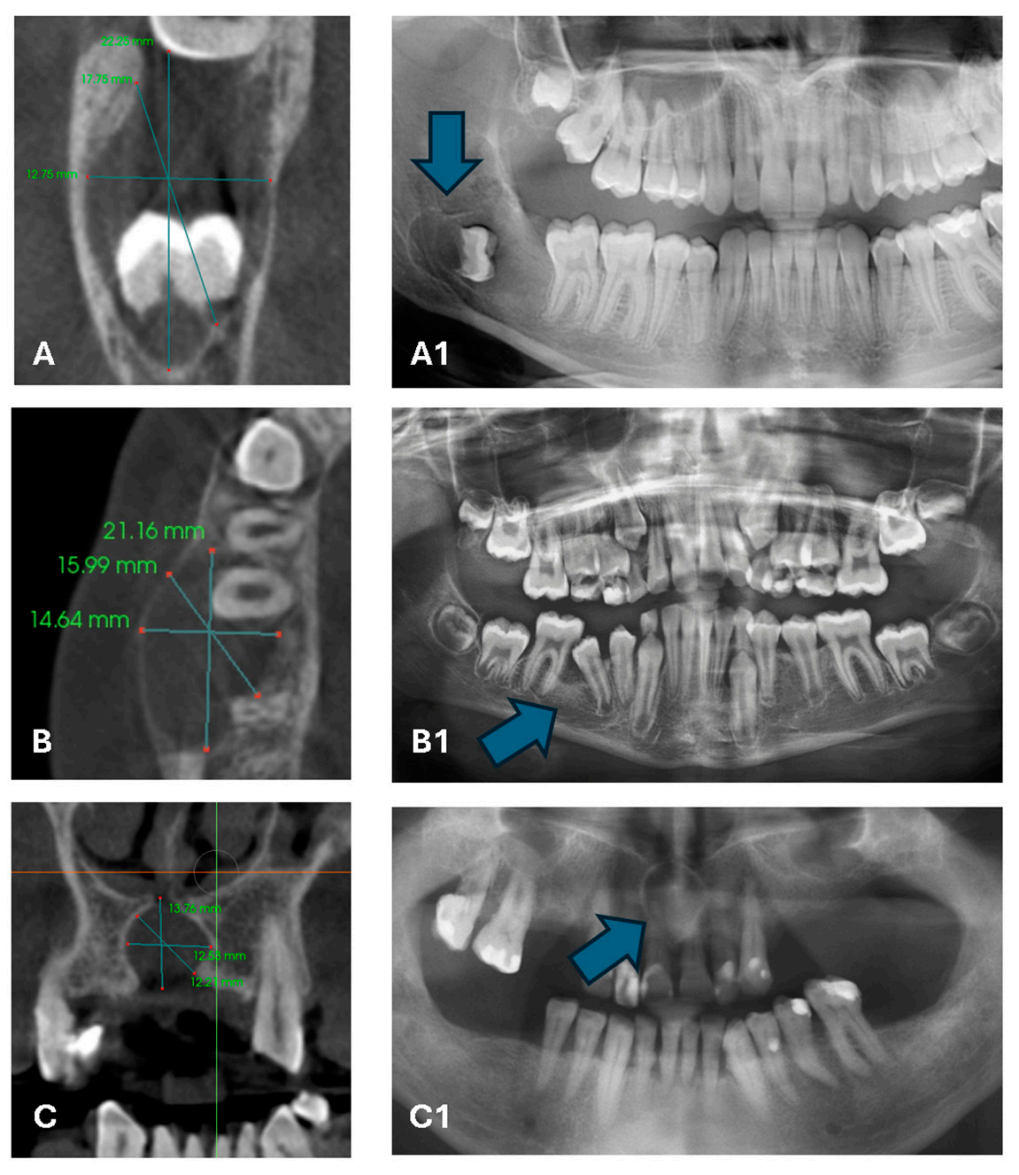
Representative imaging features of odontogenic cysts on cone-beam computed tomography (CBCT) and panoramic radiography. (**A**,**A1**) Dentigerous cyst: (**A**) CBCT showing a well-defined radiolucent lesion associated with the crown of an impacted tooth; (**A1**) corresponding panoramic radiograph. (**B**,**B1**) Odontogenic keratocyst: (**B**) CBCT demonstrating a unilocular radiolucency with characteristic dimensions; (**B1**) corresponding panoramic radiograph. (**C**,**C1**) Radicular cyst: (**C**) CBCT revealing a periapical radiolucent lesion; (**C1**) corresponding panoramic radiograph.

The diagnostic performance of human observers in the present study is consistent with previously reported limitations of radiographic evaluation of odontogenic cysts. Although clinicians achieved a high sensitivity of 88% for lesion detection, their ability to accurately differentiate between cyst subtypes remained limited, with an overall balanced accuracy of approximately 45%. This pattern—high detection but low specificity—aligns with prior observations that radiologic differentiation of jaw cystic lesions is constrained by substantial overlap in imaging features. Borghesi [6] noted that the radiographic features of odontogenic keratocysts are not pathognomonic, while Devenney-Cakir et al. [7] emphasized that interpretation of mandibular cystic lesions relies on differential, pattern-based assessment using lesion location, relationship to teeth, and secondary imaging findings. The observed intra-reader agreement of 78% in our study indicates acceptable consistency; however, it does not translate into high diagnostic accuracy, reflecting the intrinsic ambiguity of radiographic features (Figure 6).

These findings are further supported by studies comparing radiologic and histopathological diagnoses, which document clinically meaningful diagnostic discrepancy rather than consistently high overall accuracy. In mixed dentition, Mukhtar et al. [16] reported substantial pairwise discrepancy between clinical and histopathological diagnoses, particularly between dentigerous cyst and odontogenic keratocyst. Comparable limitations are evident in periapical lesion studies, where Banomyong et al. [29] found that selected radiographic features, such as involvement of multiple teeth, were associated with histopathological diagnosis, underscoring the limited discriminatory value of routine imaging alone. In the context of dentigerous cysts, the relationship to the tooth crown may aid interpretation, but variability in presentation can broaden the differential diagnosis and complicate radiologic assessment [8,9]. Additionally, the high prevalence of radicular cysts and granulomas among biopsied radiolucent jaw lesions may shape diagnostic expectations in routine practice [18].

Overall, the relatively low specificity observed in our cohort is therefore in line with the literature and highlights the inherent limitations of human visual assessment in this domain, supporting the need for adjunctive quantitative approaches such as radiomics to improve diagnostic precision.

### 4.10. Strengths and Limitations

A major strength of this study is the use of a well-defined, histopathologically confirmed cohort, ensuring reliable ground truth for all lesion types. The analysis was conducted using a fully pre-specified pipeline with strict separation of training and testing within LOOCV, minimizing information leakage and enhancing methodological transparency. In addition, the study incorporates a comprehensive radiomic framework, combining agnostic texture features with morphological and perilesional descriptors, which allowed identification of complementary sources of discriminative information. The inclusion of both mask-based and bounding-box feature extraction strategies further strengthens the analysis by demonstrating the added value of contextual information. Finally, the use of interpretable models (logistic regression, linear SVM) supports clinical applicability and facilitates understanding of feature contributions.

However, several limitations should be acknowledged. The most important are the relatively small sample size (*n* = 63) and class imbalance across cyst types (e.g., predominance of OKC vs. smaller OC-NOS group), which limit statistical power and likely contributed to the lack of significance in some pairwise comparisons. This imbalance may also introduce bias in model performance despite the use of balanced accuracy. Second, the use of single-center data and a single imaging modality (panoramic radiography) may restrict generalizability, particularly given known variability in acquisition parameters and image quality. Third, manual segmentation introduces potential observer-dependent variability, although it ensures precise lesion delineation. In addition, the OC-NOS group was defined by exclusion and is likely histologically heterogeneous; classification results involving this category should be interpreted with particular caution, as apparent separability may reflect its residual, catch-all composition rather than a coherent lesion-specific signal. Given these constraints, comparisons involving the smallest groups—particularly the eight-case OC-NOS group—carry wide confidence intervals and should be regarded as exploratory. The cohort size also limits the statistical power to establish the incremental value of the perilesional and morphological features over lesion size; this increment, although consistent, did not reach significance. Finally, structured annotations of lesion location and relationship to adjacent or impacted teeth were not consistently available in this retrospective cohort; incorporating such semantic anatomical descriptors alongside radiomic features is a key direction for future work.

Additionally, the study relies on 2D imaging, which may not fully capture the three-dimensional structure of lesions, potentially limiting feature expressiveness compared to CBCT-based approaches. Although CBCT offers three-dimensional information and superior assessment of lesion extent, it entails a higher radiation dose, greater cost, and more limited availability; the present study deliberately targeted panoramic radiography as a low-cost, widely accessible modality, and extension of the radiomic pipeline to CBCT is a natural next step. In addition, because the cohort was fully anonymised, patient demographic variables such as age and sex were not consistently available, which limits clinico-demographic correlation of the imaging findings. While LOOCV is appropriate for small datasets, it may still yield optimistic variance estimates, and the absence of external validation limits conclusions regarding real-world performance. Finally, although radiomic features were informative, no single feature achieved statistical significance after correction, reflecting both limited sample size and intrinsic overlap between lesion types. A limitation of the present study is the absence of odontogenic tumors in the analyzed cohort, which restricts direct evaluation of cyst–tumor differentiation. Future studies should include neoplastic lesions to validate the clinical applicability of radiomics-based approaches in cyst–tumor differentiation.

Overall, these limitations highlight the need for larger, balanced, multi-center datasets, standardized imaging protocols, and external validation, as well as potential integration with advanced AI methods to improve robustness and clinical applicability. Because panoramic image acquisition, image quality, and lesion prevalence vary across centers, independent external validation in multi-center cohorts is a prerequisite before the generalizability—or any clinical utility—of the present models can be assessed. The current single-center findings should accordingly be regarded as internal, proof-of-concept evidence only.

## 5. Conclusions

This study demonstrates that radiomics-based analysis of panoramic radiographs enables non-invasive differentiation of odontogenic cysts, with moderate diagnostic performance that should be regarded as exploratory and hypothesis-generating rather than clinically definitive. Pairwise classification achieved balanced accuracies up to 0.838, while multiclass classification reached 0.487, indicating that radiomic features can capture relevant lesion-specific information despite substantial overlap between cyst types.

Among feature groups, texture-based radiomics provided robust baseline discrimination, while the integration of morphological and perilesional features significantly improved performance, highlighting the importance of contextual and spatial information beyond the lesion interior. The strong contribution of perilesional descriptors suggests that surrounding bone characteristics and lesion extent are critical components of imaging-based differentiation.

However, the limited separability observed in clinically similar entities, particularly involving odontogenic keratocysts, underscores the intrinsic limitations of radiographic imaging alone and reflects the biological overlap between cyst subtypes. These findings are consistent with previous radiomics studies and reinforce the need for multimodal diagnostic strategies.

In comparison to more complex AI frameworks, the proposed approach offers a transparent, interpretable, and data-efficient solution, suitable for smaller clinical datasets. Future work should focus on larger, multi-center cohorts, external validation, and integration with advanced machine learning or deep learning models, to further assess diagnostic accuracy. External validation in independent, multi-center cohorts will be required before radiomics-based differentiation of odontogenic cysts could be considered as a complementary, supportive tool in clinical workflows.

## Figures and Tables

**Table 1:** Patient and lesion characteristics.

Class	Cases (*n*)	Cyst ROIs	Anatomical ROIs	Total ROIs	Cyst Size, Median (mm^2^)
OKC (odontogenic keratocyst)	33	36	19	55	168.9
RC (radicular cyst)	12	12	4	16	385.3
DC (dentigerous cyst)	10	11	3	14	181.1
OC-NOS (odontogenic cyst)	8	8	3	11	88.6
**Total**	**63**	**67**	**29**	**96**	**—**

OKC = odontogenic keratocyst; RC = radicular cyst; DC = dentigerous cyst; OC-NOS = odontogenic cyst. Cyst size is reported as the median cross-sectional area (mm^2^) of the largest segmented component per patient.

**Table 2:** Best-performing feature set per pairwise comparison (logistic regression, LOOCV).

Comparison	Best Feature Set	Bal. Acc.	95% CI	Sensitivity	Specificity	*p* (perm.)
DC vs. OC-NOS	Semantic (bbox)	0.838	0.662–1.000	0.875	0.800	0.004 **
OKC vs. OC-NOS	Perilesional (bbox)	0.752	0.564–0.922	0.625	0.879	0.018 *
RC vs. DC	Agnostic (bbox)	0.725	0.542–0.900	0.700	0.750	0.056
RC vs. OC-NOS	Agnostic (mask)	0.708	0.500–0.896	0.750	0.667	0.050 *
OKC vs. DC	Sem+Peri (bbox)	0.644	0.479–0.809	0.500	0.788	0.108
OKC vs. RC	Sem+Peri (bbox)	0.602	0.447–0.758	0.417	0.788	0.196

Bal. Acc. = balanced accuracy; CI = bootstrap 95% confidence interval (2000 stratified resamples); *p* (perm.) = permutation test *p*-value (500 permutations, primary model). * *p* < 0.05; ** *p* < 0.01.

**Table 3:** Ten highest-ranked radiomic features by average cross-pair AUC (mask-mode cyst ROIs).

Feature	Set	Description	Avg. AUC	Avg. d
extent_y_norm	Perilesional	Vertical extent normalised to image height	0.730	0.872
original_glrlm_RunLengthNonUniformity	Agnostic	GLRLM run-length non-uniformity	0.717	0.691
original_glszm_SizeZoneNonUniformity	Agnostic	GLSZM size-zone non-uniformity	0.717	0.691
original_ngtdm_Busyness	Agnostic	NGTDM busyness (texture heterogeneity)	0.711	0.822
original_ngtdm_Coarseness	Agnostic	NGTDM coarseness (texture fineness)	0.703	0.750
extent_x_norm	Perilesional	Horizontal extent normalised to image width	0.689	0.633
boundary_gradient_mean	Semantic	Mean gradient magnitude at lesion boundary	0.673	0.648
original_firstorder_10Percentile	Agnostic	10th percentile of intensity histogram	0.665	0.652
boundary_gradient_p90	Semantic	90th percentile of boundary gradient	0.663	0.537
original_firstorder_Median	Agnostic	Median intensity	0.661	0.541

AUC and Cohen’s d are averaged across all six pairwise class comparisons. No individual feature attained Benjamini–Hochberg significance; values are presented for exploratory rank ordering only and should not be interpreted as confirmatory. Because no feature survived correction, per-feature corrected *p*-values are not tabulated.

**Table 4:** Four-class multiclass classification results (logistic regression, LOOCV).

Feature Set	Strategy	Bal. Acc.	95% CI	*p* (perm.)
Sem+Peri (bbox)	Direct	0.487	0.352–0.621	0.002 **
Sem+Peri (mask)	Direct	0.452	0.317–0.584	0.012 *
Agnostic (bbox)	Direct	0.416	0.272–0.559	0.016 *
Semantic (bbox)	Direct	0.406	0.262–0.541	0.036 *
Sem+Peri (bbox)	Hierarchical	0.417	0.277–0.558	—
Sem+Peri (mask)	Hierarchical	0.433	0.308–0.563	—
**Chance level**	**—**	**0.250**	**—**	**—**

Bal. Acc. = balanced accuracy; CI = bootstrap 95% confidence interval (2000 stratified resamples). Permutation *p*-values (500 permutations) are reported for the logistic regression primary model under the direct strategy only. * *p* < 0.05; ** *p* < 0.01. Chance level = 0.250.

**Table 5:** Reader-specific diagnostic performance and agreement.

Reader	Balanced Accuracy	Sensitivity/Specificity, One-vs-Rest *	Cohen’s κ, Intra-Observer
Radiologist 1	0.48	0.48/0.83	0.70
Radiologist 2	0.46	0.46/0.82	0.66
Dentist 1	0.44	0.44/0.81	0.63
Dentist 2	0.42	0.42/0.80	0.59
Interobserver κ, all readers	—	—	0.30

* Sensitivity and specificity were computed one-vs-rest and macro-averaged across the four diagnostic categories; macro-averaged sensitivity is therefore numerically identical to balanced accuracy. Readers assessed the same four-class task as the radiomics model. No formal statistical comparison between readers and models was performed.

## Data Availability

All data supporting the findings of this study are available from the corresponding author upon reasonable request.

## Abbreviations

The following abbreviations are used in this manuscript:

AI	artificial intelligence
AUC	area under the curve
CBCT	cone beam computed tomography
CI	confidence interval
DC	dentigerous cyst
GLCM	grey-level co-occurrence matrix
GLDM	grey-level dependence matrix
GLRLM	grey-level run-length matrix
GLSZM	grey-level size-zone matrix
LOOCV	leave-one-out cross-validation
ML	machine learning
NGTDM	neighborhood gray-tone difference matrix
OC-NOS	odontogenic cyst, not otherwise specified
OKC	odontogenic keratocyst
RC	radicular cyst
ROI	region of interest
SVM	support vector machine
